# Supplementary material for: The interactome of intact mitochondria by cross-linking mass spectrometry provides evidence for coexisting respiratory supercomplexes
Source: Mol Cell Proteomics. 2017 Dec 8;17(2):216–32. doi: 10.1074/mcp.RA117.000470 (PMC5795388; doi:10.1074/mcp.RA117.000470)
Supplement: Supplemental Data [file supp_RA117.000470_133922_0_supp_23978_fzffwf.zip › spectra_annotation/mito_spectra_annotation/146-1-1-1-13-1.pdf]

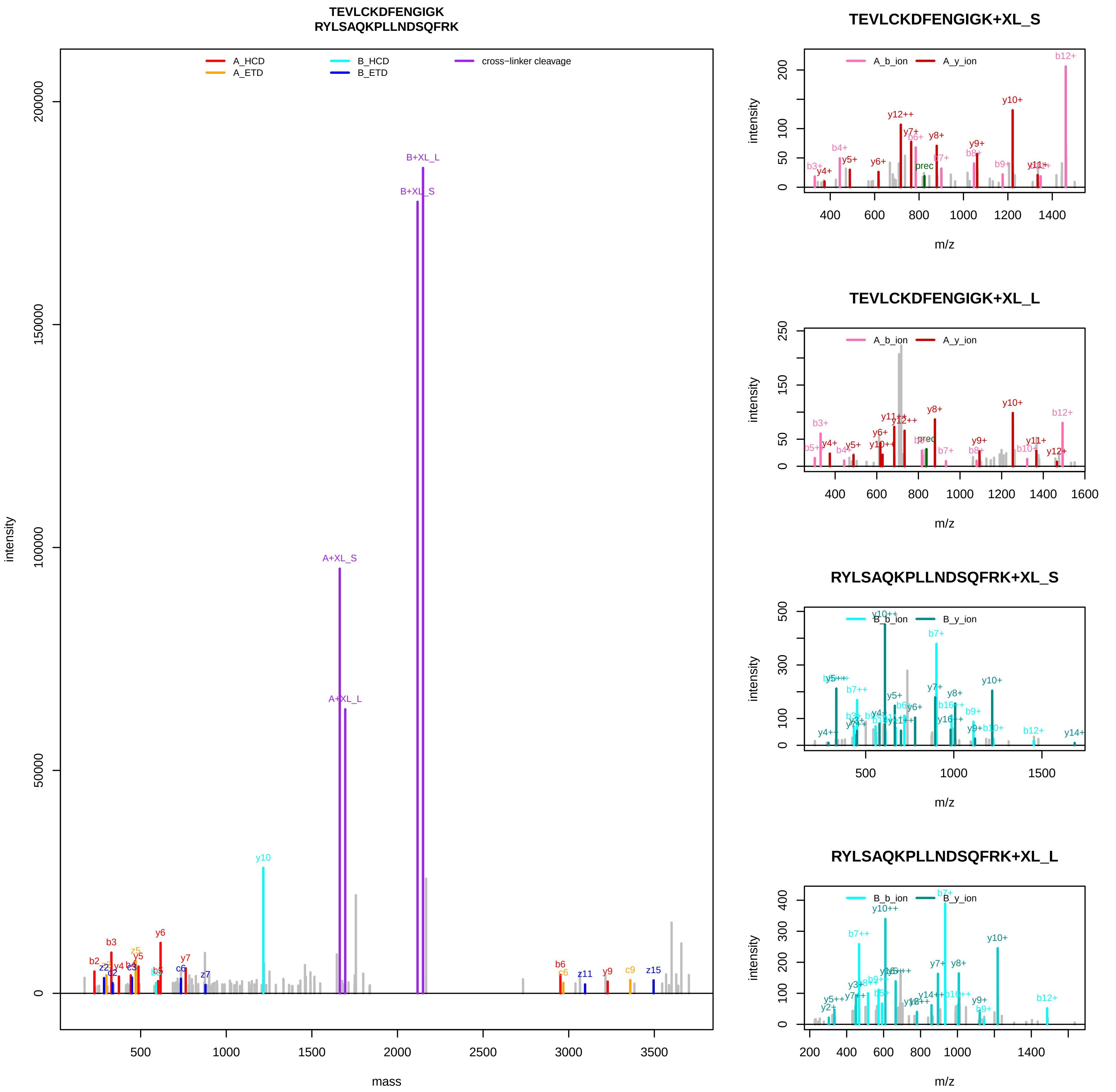
TEVLCKDFENGIGK
RYLSAQKPLLNDSQFRK
A_HCD
A_ETD
B_HCD
B_ETD
cross−linker cleavage
B+XL_L
B+XL_S
A+XL_S
A+XL_L
y10
b2
b3
y4
y5
y6
y7
c6
z7
b6
c6
z11
y9
c9
z15
intensity
mass
TEVLCKDFENGIGK+XL_S
A_b_ion
A_y_ion
b12+
y10+
y12++
prec
m/z
TEVLCKDFENGIGK+XL_L
RYLSAQKPLLNDSQFRK+XL_S
B_b_ion
B_y_ion
y10++
b7+
y7+
y8+
RYLSAQKPLLNDSQFRK+XL_L
b7++
b12+
